# Complexation of Olive Protein with Soluble Dietary Fibers: A Way to Improve the Functional Properties of Proteins and Efficiently Utilize Olives

**DOI:** 10.3390/foods13162563

**Published:** 2024-08-16

**Authors:** Yan Xiang, Yumin Chi, Qiang He, Lirong Jia, Wenxue Zhang, Yi Dong

**Affiliations:** Healthy Food Evaluation Research Center, College of Biomass Science and Engineering, Sichuan University, Chengdu 610065, China; xiangyanxy2024@163.com (Y.X.); chiyumin@teway.cn (Y.C.); heq361@163.com (Q.H.); jialirong@scu.edu.cn (L.J.); zhangwenxue@scu.edu.cn (W.Z.)

**Keywords:** biopolymers, complex coacervation microstructure, emulsifying properties, olive protein

## Abstract

High-value resources beyond oil extraction for the olive industry need to be developed due to increased olive production. Soluble dietary fibers (SDFs) and olive proteins (OPIs) are important components of olives. However, the commercial production process partially damages OPIs’ emulsifying and foaming properties. Thus, the preparation of SDF-OPI complexes would help protect and even improve the emulsifying and foaming properties. The effects of pH and thermal–ultrasonic treatment on the complexation were explored, which showed that the SDF-OPI complexes prepared at pH 5 exhibited superior solubility (*p* < 0.05). SDF addition noticeably improved OPI thermal stability, emulsifying properties, and foaming properties. Moreover, the complexes prepared by thermal–ultrasonic treatment exhibited higher emulsion stability and lower emulsification activity than those prepared without thermal–ultrasonic treatment. In the acidic system, the electrostatic interaction was considered the main driving factor, assisted by the hydrophobic interaction. Additionally, after thermal–ultrasonic treatment, the covalent binding was observed by infrared spectroscopy. These results revealed the interaction mechanism between SDF and OPI, and the complexes significantly enhanced the functional properties of OPI. This study provides a reference for the high-value utilization of olives, thus broadening their potential uses in the food sector and beyond.

## 1. Introduction

The olive (*Olea europaea* L.) is an oil crop in Asia Minor [1]. It grows mainly in the Mediterranean area, including Greece, Italy, and Spain [2]. The global production of table olives surpassed approximately 2.7 million tons during the 2020–21 season according to the International Olive Council (IOC) [3]. Today, in order to extend the olive-related industry chain, high-value resources besides oil extraction are urgently needed in the olive industry as olive production increases.

Olive pomace contains 1–4% protein, 5–11% fat, 10–17% ash, and 3.1–3.8 g gallic acid equivalents/100 g of phenolic content (dry weight) [4]. The soluble dietary fiber (SDF) from olive pulp mainly consists of xylose [5]. Compared to insoluble dietary fibers (IDFs), SDF holds water and adsorbs substances better [6]. SDF can also absorb cholesterol, lower postprandial blood glucose levels, and can even be used as a prebiotic for gut microbial flora [7]. As a natural foam stabilizer and emulsifier, the proteins of the plant origin are relatively cheap, which are worth further developing from both the nutritional and functional perspectives [8]. The seed storage protein, formed during seed development and mainly stored in specialized storage tissues (e.g., cotyledons and endosperm), is the main protein (OPI) in olive kernels [9,10]. However, the commercial production process will partially damage OPI’s emulsifying and foaming properties [11]. Therefore, the proper functionalization of OPI is necessary.

Attaching polysaccharides to proteins is a well-recognized strategy to improve their solubility, emulsifying properties, foaming properties, and antioxidant stability [12,13,14]. Protein–polysaccharide complexes are often used as emulsifiers or emulsion stabilizers, which can substantially improve food products’ sensory and textural structure [15]. Ultrasonic emulsification and homogeneous emulsification are two common methods for preparing emulsions. Previous research has explored the effects of the complexation of various polysaccharides and proteins on functional attributes to extend their applications within foods and other products [16,17,18]. The complexation is mainly governed by pH [19]. Moreover, some processing technologies, including sonication and heating, have been demonstrated to amplify the reactions involved in protein modification, which can be a candidate method for promoting protein and polysaccharide complexation [20]. However, there is little information about the interaction between SDF and OPI from olives and the further changes in their properties. 

This study used the SDF from olive pulp and OPI from olive kernels to prepare complexes. The impact of pH and thermal–ultrasonic treatment on the complexation of SDF with OPI was examined, and the changes in the thermal properties, emulsifying properties, and foaming properties of SDF-OPI complexes were analyzed. Finally, the interaction mechanism of the complex was determined. This study aims to establish a new complex for olive protein and dietary fiber and to investigate its potential applications.

This study could help to reveal the potential of olives for producing biopolymers with favorable properties, thus increasing the added value of olives.

## 2. Materials and Methods

### 2.1. Materials

Fresh olives were provided by Sichuan Olive Branch Agricultural Technology Co., Ltd. (Chengdu, China). Sodium hydroxide (NaOH) and sodium chloride (NaCl) were of analytical grade and purchased from Jinshan Chemical Reagent Co., Ltd. (Chengdu, China). Urea and dodecyl sodium sulfonate (SDS) were purchased from Adamas Reagent Co., (Shanghai, China) and had a purity of ≥99.7%.

### 2.2. Preparation and Characterization of SDF

#### 2.2.1. Preparation of SDF

The SDF was prepared as previously described [21]. SDF in olive pulp was extracted by alkaline extraction and then dried using hot air at 50 °C. The content of SDF after extraction was 92.89%.

#### 2.2.2. X-ray Diffraction (XRD)

The SDF was scanned using a PANalytical X-ray diffractometer (PANalytical B.V., Almelo, The Netherlands). Each sample was scanned between 2θ = 5–50° with a step size of 0.02° at a scanning rate of 2°/min [22].

### 2.3. Preparation and Characterization of OPI

#### 2.3.1. Preparation of OPI

The OPI was extracted from olive kernel followed by Miranda et al. [23]. The dehydrated and defatted kernel powder was dispersed in deionized water (100 g/L) and left to soak for 2 h. Then, the mixture was adjusted to pH 9.0 with NaOH (1 M) and stirred at 50 °C for 2 h. After filtration, the filtrate was adjusted to the isoelectric point (pH 4.5–5.0) with citric acid (1 M) and kept for 1 h. Finally, the filtrate was centrifuged at 13,304× *g* for 20 min using a 1736R large capacity high-speed refrigerated centrifuge (Gene Technology Co., Ltd, Wuhan, China) and the residue was transferred to a SCIENTZ-10N freeze dryer (Scientz Biotechnology Co., Ningbo, China) for freeze-drying to obtain the OPI with a content of 86.79%.

#### 2.3.2. Characterization of the Secondary Structure

The circular dichroism (CD) measurement was conducted on a Chirascan Plus V100 CD spectrometer (Applied Photophysics, Surrey, UK) according to Bilir et al. [24]. The protein solution (0.2 mg/mL) was prepared with PBS buffer (pH 7.0, 10 mM) and loaded into quartz cells at a consistent temperature of 25 °C. The scanning range was 190–250 nm in 1 nm steps.

### 2.4. Preparation of SDF-OPI Complexes

The SDF and OPI were compounded as previously described [20,25]. OPI (0.18 g) and SDF (0.18 g) were dissolved in distilled water (6 mL) in two stoppered tubes, respectively, and shaken for 1 h at room temperature. Then, two solutions (1:1, *v*/*v*) were mixed, and 6 mL of citrate–phosphate buffer of different pH (10 mM, pH 3.0, 4.0, 5.0, 6.0, 7.0) was added. The solution was dissolved in a stoppered conical flask by a SHA-C water bath thermostatic oscillator (Jintan Science Analysis Instrument Co., Ltd., Changzhou, China) for 2 h at room temperature. Then, half of the solution was taken through freeze-drying with a SCIENTZ-10N freeze-dryer, to obtain the SDF-OPI complex without thermal–ultrasonic treatment (hereinafter referred to as the untreated SDF-OPI complex). The solution’s other half continued to react at 80 °C for 1 h in the SHA-C water bath thermostatic oscillator. After being cooled in running water, it was ultrasonicated on a SCIENTZ-IID ultrasonic cell pulverizer (Scientz Biotechnology Co., Ningbo, China) using an amplitude of 35 μm at 360 W for 10 min. The SDF-OPI complex powder was obtained by freeze-drying at −40 °C in a vacuum of 10 Pa for 36 h with the SCIENTZ-10N freeze-dryer and stored at 4 °C until further analysis.

### 2.5. Effect of pH during Preparation on the Complexes

#### 2.5.1. ζ-Potential and Particle Size

The ζ-potential and particle size were measured by a nano-ZS particle size potentiostat (Malvern Panalytical Co., Worcestershire, UK). The SDF-OPI complex solutions (1 mg/mL) were filtered through a 0.45 µm Millipore membrane. The phosphate buffer (10 mM, pH 3.0, 4.0, 5.0, 6.0, 7.0) was used as the dispersant.

#### 2.5.2. Turbidity

The turbidity of the SDF, OPI, and SDF-OPI complex was assessed by measuring the absorbance at 600 nm (UV-6000PC, Shanghai Metash Instruments Co., Shanghai, China) according to Dong et al. [26]. The phosphate buffer (10 mM, pH 3.0, 4.0, 5.0, 6.0, 7.0) was used as the dispersant. The ultrapure water was used as a control.

#### 2.5.3. Solubility

The solubility of the SDF, OPI, and SDF-OPI complex was measured according to Rezaei et al. [27]. The phosphate buffer (10 mM, pH 3.0, 4.0, 5.0, 6.0, 7.0) was used as the dispersant. The solubility was expressed in percentages.

#### 2.5.4. Intrinsic Fluorescence Analysis

The fluorescence emission spectra (300–450 nm) of the SDF, OPI, and SDF-OPI complex solution (1 mg/mL) were recorded with an F-7000 fluorophotometer (Hitachi Ltd., Tokyo, Japan) at a fixed excitation wavelength of 270 nm [28]. The phosphate buffer (10 mM, pH 3.0, 4.0, 5.0, 6.0, 7.0) was used as the dispersant.

### 2.6. Fourier-Transform Infrared Spectroscopy (FTIR)

Complex powders were mixed with potassium bromide powder at a ratio of 100:1 and then ground and compressed to form disc-shaped pellets. The FTIR was collected at 25 °C on a NEXUS 670 infrared spectrometer (Thermo Scientific, Madison, WI, USA) under the wavenumber range of 4000 to 400 cm^−1^.

### 2.7. Scanning Electron Microscope (SEM)

The samples were attached to a double-sided carbon tape on a cylindrical aluminum mount and then sputter-coated with gold. The microstructure was examined with a SU5000 scanning electron microscope (Hitachi Ltd., Tokyo, Japan).

### 2.8. Differential Scanning Calorimetry (DSC)

The thermal properties were determined using an EXSTAR6000 thermal analyzer (Hitachi Ltd., Tokyo, Japan). The samples (2 mg) in crucibles were heated from 30 to 200 °C at a rate of 10 °C/min with a nitrogen flow rate of 20 mL/min. A blank crucible was used as a control.

### 2.9. Determination of Electrostatic Interactions, Hydrophobic Interactions, and Hydrogen Bonding

SDF-OPI complex emulsions were prepared by adding NaCl (0.20 M), urea (0.10 M), and SDS (1 g/L) to SDF-OPI complex solutions (3.0%, *w*/*v*) with different pHs (3, 4, 5, 6, 7). The SDF-OPI emulsion without these additions was used as a control. The absorbance of the emulsion containing SDS or urea was measured at 600 nm to determine the turbidity [29]. The ζ-potential of the emulsion containing NaCl was determined using a nano-ZS particle size potentiostat.

### 2.10. Emulsifying Properties

#### 2.10.1. Preparation of Ultrasonic Emulsions and Homogeneous Emulsions

According to the 1:1 mass ratio of SDF to OPI in the preparation of the complexes, the SDF-OPI complex emulsion (1:1 mass ratio, 3.0% *w*/*v* total biopolymer concentration), the untreated SDF-OPI complex emulsion (3.0%, *w*/*v*), the SDF emulsion (1.5%, *w*/*v*), and the OPI emulsion (1.5%, *w*/*v*) were prepared using citrate–phosphate buffer (10 mM, pH 5.0) [30,31]. After adding the soybean oil (10.0%, *v*/*v*), the ultrasonic emulsions were prepared using an ultrasonic cell pulverizer at 300 W for 4 min, while the homogeneous emulsions were prepared using an FJ300-SH high-speed homogenizer (Shanghai HU Analytical Industry Co., Shanghai, China) at 10,000 rpm for 2 min [32].

#### 2.10.2. Determination of Emulsification (EAI) and Emulsion Stability (ESI)

Amounts of 40 μL of the emulsion were added into 4 mL of SDS solution (1 mg/mL) at 0 min and 10 min after the preparation of emulsion, and the absorbance was measured by a Synergy H1 multifunctional microplate detector (BioTek Instruments, Inc., Winooski, VT, USA) at 500 nm. The *EAI* and *ESI* were calculated by Equations (1) and (2), respectively [33]

(1) $$EAI\left(m^{2}/g\right)=\frac{2\times 2.303\times A_{0}\times D}{C\times \left(1-\emptyset \right)\times 10^{4}},$$

(2) $$ESI\left(\%\right)=\frac{A_{10}}{A_{0}}\times 100,$$

where ∅ is the oil-phase fraction (10% in this study), *C* is the concentration of the original sample solution (g/mL), and *D* is the dilution factor. *A*_0_ and *A*_10_ are the absorbances at 0 and 10 min.

#### 2.10.3. Storage Stability of Emulsions

The SDF-OPI complex emulsion, untreated SDF-OPI complex emulsion, SDF emulsion, and OPI emulsion were kept at room temperature for 12 days and the changes in the EAI and ESI were tested.

#### 2.10.4. Ionic Stability of Emulsions

NaCl (with a final concentration of 0.2 M) was, respectively added to the SDF-OPI complex emulsion, untreated SDF-OPI complex emulsion, SDF emulsion, and OPI emulsion prepared by sonication at 300 W for 4 min, and their EAI and ESI were determined [34]. The emulsion without NaCl was used as a control.

#### 2.10.5. Thermal Stability of Emulsions

The SDF-OPI complex emulsion, untreated SDF-OPI complex emulsion, SDF emulsion, and OPI emulsion were prepared by sonication at 300 W for 4min and then placed in boiling water for 20 min. After cooling to room temperature, the change in the EAI was determined. The control was the emulsion left at room temperature for 20 min [35].

### 2.11. Foaming Properties

The foaming properties were measured following Alavi et al. [36]. Each sample solution (50 mL) was processed in an FJ300-SH high-speed dispersing homogenizer at 10,000 rpm for 2 min. The foam volume was measured after keeping it for 30 min. Foamability (*FA*) and foam stability (*FS*) were calculated by Equations (3) and (4)

(3) $$FA\left(\%\right)=\frac{V_{0}}{V_{L}}\times 100,$$

(4) $$FS\left(\%\right)=\frac{V_{30}}{V_{0}}\times 100,$$

where *V*_0_ is the initial foam volume, *V_L_* is the volume of the solution, and *V*_30_ is the foam volume at 30 min.

### 2.12. Statistical Analysis

All experiments were prepared in triplicates with freshly prepared samples. Data were analyzed by analysis of variance (ANOVA) and differences between mean values were determined by Fisher’s least significant difference (LSD) at a *p* < 0.05 significance level using IBM SPSS Statistics 24 software.

## 3. Results

### 3.1. Effects of pH during Preparation on the SDF-OPI Complexes

#### 3.1.1. ζ-Potential and Size Analysis

**Figure 1:**
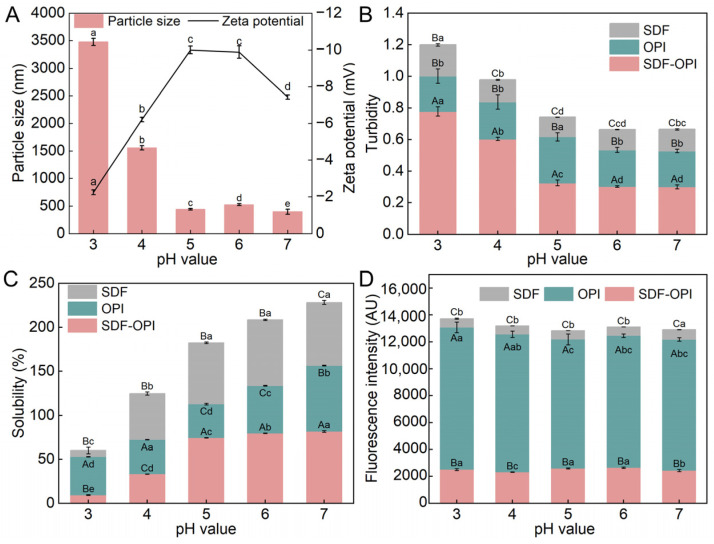
Effects of pH during preparation on the complexation of soluble dietary fiber (SDF) with protein (OPI). (**A**) The ζ-potential and average particle size of SDF-OPI complex; the (**B**) turbidity, (**C**) solubility, and (**D**) fluorescence intensity of SDF, OPI, and SDF-OPI complex. ^A,B,C^: Different uppercase letters indicate significant differences among substances; ^a,b,c,d,e^: different lowercase letters indicate significant differences among pH values (*p* < 0.05). Data represent the mean of three experiments.

The ζ-potential is considered an indicator of electrostatic interactions between colloidal particles. As seen in Figure 1A, the complexes prepared at pH 5 had the lowest ζ-potential (*p* < 0.05). Studies have shown that one major driver of the complex formation between proteins and dietary fibers is electrostatic adsorption [37]. Typically, dietary fibers are negatively charged, whereas proteins can have different charges depending on pH value. When prepared at pH 6, a small portion of OPI was positively charged and bound to the negatively charged SDF, causing the protein structure to stretch out. The protein residues with negative charge were exposed, which might cause the ζ-potential to drop [38]. When prepared at pH < 5, the isoelectric point of the OPI had been crossed, more positively charged proteins were absorbed into the negatively charged SDF, and the ζ-potential increased. 

The particle sizes of SDF-OPI prepared at different pH are summarized in Figure 1A. The particle size increased significantly from pH 5 to 3 (*p* < 0.05), consistent with the changing trend of ζ-potential. The formation of complexes between SDF and OPI increased the particle size [39]. The components were less likely to agglomerate at pH 5 because of the electrostatic repulsion, which made the existence of a smaller particle size and a more stable complex solution possible.

#### 3.1.2. Turbidity

The turbidity of the complex solution can describe the binding of the complexes and help visualize how the complexes will appear in food systems [26]. As shown in Figure 1B, as the pH during preparation increased, the turbidity of the SDF gradually decreased and then stabilized, and the overall turbidity was lower than that of OPI. This was due to the negative charge on the surface of SDF being neutralized in the strongly acidic environment. Therefore, the electrostatic repulsive force decreased, resulting in lower molecular dispersion. The turbidity of OPI reached the peak at pH 5, which was known as the isoelectric point of OPI. This observation was made during the change in turbidity of most polysaccharides and proteins [26]. On the other hand, the turbidity of the SDF-OPI complex decreased as the pH during preparation increased and stabilized when the pH approached neutrality. Turbidity is positively correlated with the particle size, which can be found in Figure 1A,B. In a strongly acidic environment, SDF could not provide a sufficient electrostatic barrier to prevent aggregation between proteins [40]. When prepared at pH ≥ 5, the turbidity of the complex system was lower than the total turbidity of SDF and OPI, which indicated better dispersion.

#### 3.1.3. Solubility

The solubilities of SDF-OPI complexes prepared at different pH values were also determined (Figure 1C). When prepared at pH < 5, the solubility of the SDF-OPI complex was worse than that of the original OPI, which may be because the complex formed at this pH had a larger size and was deposited more easily. This finding is consistent with previous studies, which reported that proteins and anionic polysaccharides tend to form insoluble complexes when the pH value is lower than the isoelectric point of proteins [41]. Moreover, the solubility of the SDF-OPI complexes prepared in the pH range of 5–7 was higher than that of the OPI, indicating that the SDF-OPI complexes could improve the solubility of OPI. The complex prepared at pH 5 showed the best improvement effect with an increase from 36.14% to 74.38%, which was beneficial to apply in liquid foodstuffs. Polysaccharides had spatial site-blocking and entanglement effects that could effectively inhibit the aggregation and sinking of proteins. Besides, more hydrophilic groups were presented in glycosylated proteins, which would contribute to their dispersion in aqueous solutions [27].

#### 3.1.4. Fluorescence Intensity Analysis

Certain aromatic amino acids (predominantly tryptophan) in proteins exhibit intrinsic fluorescence upon excitation at specific wavelengths and are sensitive to alterations in microenvironment and tertiary protein structure [28]. As shown in Figure 1D, the fluorescence intensities of the SDF-OPI complexes were substantially lower than those of OPI, indicating that the fluorescence burst of the OPI’s chromophores occurred during the complexation process. Intermolecular entanglement and cross-linking would cause aromatic amino acid residues to be encapsulated inside the complex, reducing fluorescence intensity [28]. The sedimentation and aggregation of SDF-OPI occurred when the pH dropped, then the chromophore inside was encased and the fluorescence intensity was lowered [42]. The SDF-OPI complex formed at pH 5 had smaller particle sizes, lower turbidity, and higher solubility. Therefore, the SDF-OPI complexes prepared at pH 5 were selected for subsequent experiments.

### 3.2. XRD Analysis of SDF

**Figure 2:**
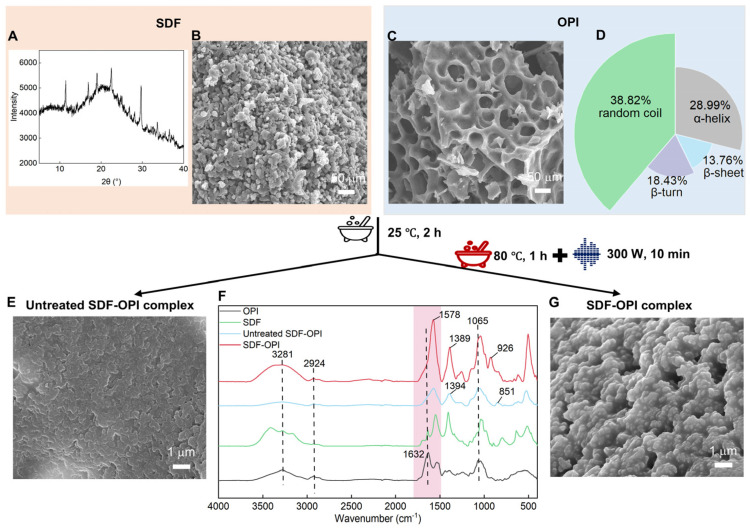
(**A**) The X-ray diffraction patterns of SDF; appearance of SDF (**B**) and OPI (**C**) under 1000× electron microscope; (**D**) secondary structure content of OPI; (**E**) appearance of the untreated SDF-OPI complex under 5000× electron microscope; (**F**) FTIR spectroscopy of OPI, SDF, untreated SDF-OPI complex, and SDF-OPI complex; (**G**) appearance of the SDF-OPI complex under 5000× electron microscope. The difference between the SDF-OPI complex and the untreated SDF-OPI complex was that thermal–ultrasonic treatment was involved in the preparation of the former.

The XRD result of SDF is listed in Figure 2A. Within the 15°–30° range, broad and low-intensity peaks suggested that SDF was a predominantly cellulose type I with an amorphous structure, coexisting with crystalline domains. It was similar to the crystal structure of water-soluble dietary fiber from corn bran [43].

### 3.3. The Secondary Structural Analysis of OPI

Circular dichroism is commonly used for the secondary structure analysis of protein. The content of each secondary structure can be obtained after calculation, and the outcomes are displayed in Figure 2D. OPI had the lowest β-folding content, which was usually regarded as related to protein stability. The lower level of β-sheet was associated with weaker protein stability. Random coils and α-helixes dominated the secondary structure, making up over 67% of the OPI’s structure, which suggested that OPI was conformationally flexible and its molecules were open and pliable [44].

### 3.4. Thermal Characterization

Thermal characterization is used to investigate structural transformation and stability during heat treatment. The denaturation temperature and enthalpy change positively correlate with thermal stability [42]. Table 1 shows that SDF exhibited greater thermal stability compared to OPI. The denaturation temperature of the SDF-OPI complex was significantly higher than that of OPI (*p* < 0.05), which indicated that the thermal stability of OPI was enhanced after being compounded with SDF. On the other hand, the enthalpy change values of the thermal–ultrasonic SDF-OPI complex were also significantly higher than those of the untreated SDF-OPI complex (*p* < 0.05). Original hydrogen bonds in SDF and OPI were broken by ultrasonic treatment, while new hydrogen bonds were created between the two, which may consequently increase the thermal stability of the complexes [42].

### 3.5. FTIR

The amide I band (1600–1700 cm^−1^, mainly corresponding to C=O bonding) and amide II band (1480–1580 cm^−1^, mainly corresponding to N-H bending and C-N stretching) can be used to analyze the changes in the secondary structure of proteins. The amide I and II band profiles of SDF-OPI resembled SDF but exhibited shifts compared to OPI: Amide I shifted from 1632 cm^−1^ to 1578 cm^−1^ and Amide II from 1535 cm^−1^ to 1389 cm^−1^ (Figure 2F). The result indicated that the complexation with SDF affected the secondary structure of the OPI and the stretching vibrations of its characteristic motifs. Previous studies reported that electrostatic interactions between the protein’s +NH^3+^ and the polysaccharide’s -COO^−^/-OH^−^ may modify the protein’s α-helix structure, subsequently influencing its amide band [45].

Compared with the untreated SDF-OPI, the SDF-OPI gave broader peaks from 3000 to 3600 cm^−1^ (mainly related to O-H bonds) (Figure 2F), which can be attributed to the stretching vibration of the N–H and O–H bonds in intermolecular hydrogen bonds [46]. In addition, shifts of the peaks and stronger absorption at 1500–1700 cm^−1^ (mainly related to C=O bonding) and 900–1100 cm^−1^ (mainly related to C-O, C-N, C-H, and N-H bonding) were also observed in SDF-OPI as compared to SDF-OPI, which may be attributed to the Maillard reaction [36,47]. Under optimal humidity and temperature, proteins and polysaccharides in a system can be covalently linked via the Maillard reaction [48]. In other words, a hydroxyl–ammonia reaction could occur between the free amino groups on the side chains of proteins (predominantly ε-amino groups on lysine) and the reducing ends of polysaccharides (predominantly hydroxyl groups). It was speculated that during processing (mainly in heat treatment), the covalent binding of OPI and SDF occurred, resulting in the deformation vibrations of C=O, N-H, and C-N bonds in the main chain of OPI, as well as the O-H, C-H, and C-O bonds on the protein side chains and SDF [15]. Moreover, the peak area near 3300 cm^−1^ in SDF-OPI increased compared to untreated SDF-OPI, likely attributed to enhanced hydroxyl and hydrogen bonding from SDF integration. It coincided with the manifestation of higher hydrophilicity and the thermal stability of SDF-OPI. 

### 3.6. SEM

SDF showed a highly granular and uneven surface with densely distributed pores and folds, which could absorb and bind other substances well (Figure 2B). The large pores on the surface of OPI (Figure 2C) may be due to numerous hydrophobic residues on the surface and strong inter-protein interactions [49]. SDF-OPI had a rough surface with tiny particles, riddled with many microscopic cavities (Figure 2G). Compared with SDF-OPI, the untreated SDF-OPI surface was relatively smooth and lamellar, with larger cavities and no obvious granularity (Figure 2E), resulting in a smaller specific surface area. The SDF-OPI prepared by thermal–ultrasonic treatment had closer interactions and uniform morphology with a larger specific surface area, which could explain its better solubility and higher reactivity. In addition, previous studies have demonstrated that thermal and ultrasonic treatments promoted modifications resulting in closer SDF-OPI interactions [20].

### 3.7. Electrostatic Interactions, Hydrophobic Interactions, and Hydrogen Bonding

**Figure 3:**
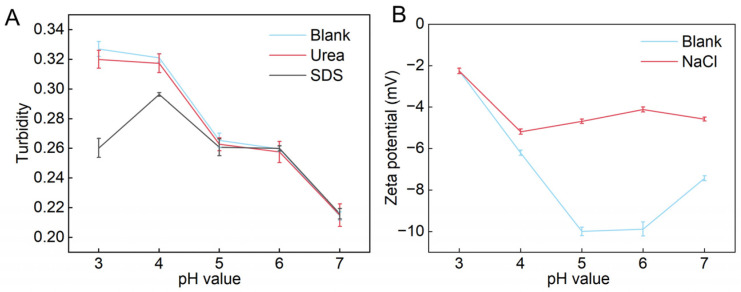
At different pH values, the effects of (**A**) urea and dodecyl sodium sulfonate (SDS) on turbidity and of (**B**) NaCl on ζ-potential of SDF-OPI complexes.

Wang et al. [29] state that urea and SDS disrupt the hydrogen bonding and hydrophobic interactions in the emulsions, respectively. For the solutions with SDS, the turbidity was insignificantly different from the blank group at pH 5–7, while at pH 3 and 4, the turbidity was significantly lower than the blank group (Figure 3A). On the other hand, pH could affect turbidity, as shown that turbidity increased significantly from pH 3 to 4, and then decreased as pH increased, with little change in the pH 5–6 range. This result indicated that the change in turbidity might be related to the hydrophobic interaction. Meanwhile, there was no significant difference between the solutions with urea and the blank group at the same pH, indicating that hydrogen bonding did not affect the turbidity of the SDF-OPI emulsion.

Wang et al. [29] found that the ionic species in NaCl interact with the ionized colloidal ions, compressing the double electric layer around the colloidal molecules, resulting in a decrease in ζ-potential. This was corroborated in our study (Figure 3B), indicating that a high concentration of salt ions in the emulsion led to electrostatic shielding. The SDF-OPI emulsion without NaCl showed the lowest ζ-potential and carried the most charge at pH 5, while the SDF-OPI emulsion containing NaCl (0.20 M) held the most charge at pH 4. This result indicated that NaCl would inhibit the formation of the complex. The electrostatic shielding from NaCl caused a relatively low charge in the system, only when the pH was lower was the emulsion able to provide enough charge to allow electrostatic adsorption between the SDF and the OPI. It could be hypothesized that as the electrostatic interaction was considered the main driving factor in the complexes, the hydrophobic interaction was assisted and the hydrogen bonding was insignificant.

### 3.8. EAI and ESI Analysis

#### 3.8.1. EAI and ESI under Different Emulsification Methods

**Figure 4:**
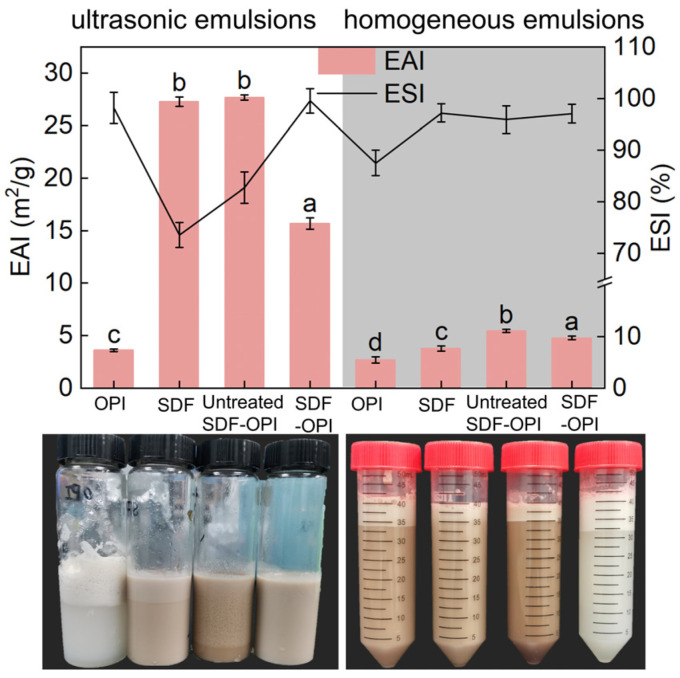
Emulsification (EAI) and emulsion stability (ESI) of ultrasonic emulsions (**left**) and homogeneous emulsions (**right**) of OPI, SDF, untreated SDF-OPI, and SDF-OPI complex. ^a,b,c,d^: Different lowercase letters indicate significant differences (*p* < 0.05).

The emulsifying properties reflect the interfacial properties for forming emulsions of the substance. The emulsifying experiments were performed by dissolving SDF, OPI, and their complexes in a citrate-phosphate buffer (pH 5) to simulate the most common acidic systems in foods. As shown in Figure 4, the EAI of OPI in both ultrasonic emulsions (3.60 m^2^/g) and homogeneous emulsions (2.69 m^2^/g) was the weakest of the four substances, mainly because its solubility was the lowest at pH 5, which made it difficult to disperse uniformly for emulsifying. SDF also showed certain emulsifying properties and the EAI in ultrasonic emulsions (27.68 m^2^/g) was significantly higher than that in homogeneous emulsions (3.77 m^2^/g) (*p* < 0.05). The porous structure of SDF can absorb oil well, and the hydrophilic groups on the surface of SDF allow it to stably disperse in water, which is beneficial to preparing oil-in-water emulsions [50]. The EAI of SDF-OPI in both ultrasonic emulsions (15.68 m^2^/g) and homogeneous emulsions (4.79 m^2^/g) was increased compared with OPI emulsions, which suggests that the compounding could enhance the emulsifying properties of OPI. During the emulsifying process, proteins and polysaccharides could interact to form a strong interfacial membrane. Polysaccharides can also prevent droplet aggregation through spatial site-blocking effect and gelation behavior, thus improving the system’s stability. That was consistent with previous research from Fan et al. [18] who observed that the protein–polysaccharide complexes could enhance the emulsion stability.

In both ultrasonic emulsions and homogeneous emulsions, the ESI of SDF-OPI was stronger than that of untreated SDF-OPI, while the EAI showed the opposite result (Figure 4). Thermal and ultrasonic treatments exposed the internal groups of SDF and OPI, reduced the particle size, and accelerated the reaction rate so that the degree of binding between SDF and OPI in the SDF-OPI was higher than that in the untreated SDF-OPI [30]. Compared to SDF-OPI, the untreated SDF-OPI complex had a smaller particle size and larger specific surface area, and the hydrophilic groups of SDF were not fully buried inside the complex, providing an emulsifying advantage. However, these free SDFs may also trigger flocculation due to evacuation, resulting in decreased ESI [31].

Comparing the two emulsification methods of ultrasonic emulsification and homogeneous emulsification horizontally, it can be seen that the EAI of ultrasonic emulsions was significantly better than that of homogeneous emulsions. Ultrasound could promote heightened breaking efficiency by facilitating the attainment of smaller droplet particle sizes. This observation was in line with the research from Liu et al. [31], which indicated that the EAI increased as the particle size of the droplets became smaller. However, the average emulsion stability of ultrasonic emulsions was less than that of homogeneous emulsions, which is attributed to the damage caused by ultrasound to the structure of the substances (mainly the SDF). This inference also supports the above point that the emulsification in untreated SDF-OPI mainly relies on free SDF.

#### 3.8.2. Storage Stability Analysis

**Figure 5:**
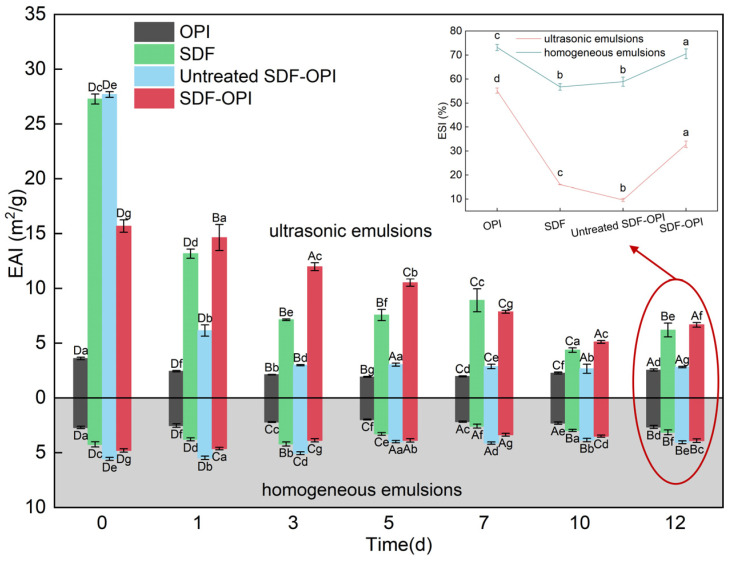
Changes in EAI and ESI of ultrasonic emulsions and homogeneous emulsions of OPI, SDF, untreated SDF-OPI, and SDF-OPI complex during 12 days of storage at room temperature. ^A,B,C,D^: Different uppercase letters indicate significant differences among storage days; ^a,b,c,d,e,f,g^: different lowercase letters indicate significant differences among substances (*p* < 0.05).

In ultrasonic emulsions stored for more than 1 day, the EAI of SDF-OPI was the highest. In homogeneous emulsions, the EAI of untreated SDF-OPI was the highest within 12 days (Figure 5). It could be inferred that emulsions prepared by different emulsification methods require suitable emulsifiers. The EAI of the ultrasonic emulsion after 12 days was higher than that of the homogeneous emulsion, while the ESI showed the opposite result (Figure 5). The SDF was broken into smaller particle-size polysaccharides or oligosaccharides by ultrasound and the specific surface area was increased, which caused the newly formed emulsion to join numerous tiny oil droplets with the high EAI. However, the destruction of its porous structure also reduced the ability to maintain stability [30]. In terms of EAI, ultrasound was better and more convenient than homogenization. Therefore, the ultrasonic process was chosen for subsequent emulsification experiments.

#### 3.8.3. Ionic Stability Analysis

**Figure 6:**
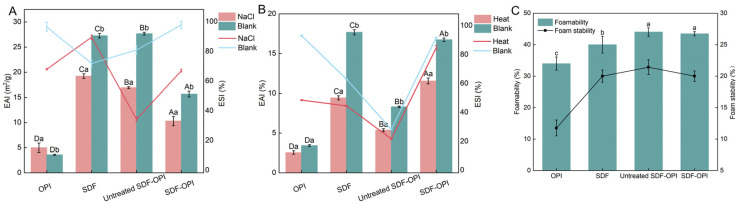
Effects of NaCl (**A**,**B**) heat treatment on EAI and ESI of ultrasonic emulsions of OPI, SDF, untreated SDF-OPI, and SDF-OPI complex emulsions. The bars represent the EAI, and the lines represent the ESI. In addition, different uppercase letters (^A,B,C,D^) indicate significant differences among substances, and different lowercase letters (^a,b,c^) indicate significant differences (*p* < 0.05) between the blank and experimental groups. (**C**) Foaming ability and stability of OPI, SDF, untreated SDF-OPI, and SDF-OPI complex (^a,b,c^: different lowercase letters indicate significant differences (*p* < 0.05) among substances).

Since emulsions are widely utilized in biomolecule delivery systems and various commercial products containing salt ions, it is crucial to evaluate the ionic stability of emulsions. The addition of NaCl (0.15–0.30 M) to ultrasonic emulsions allows for the investigation of emulsifying properties at high ionic concentrations [34]. As seen in Figure 6A, except for the OPI emulsion, the EAI of other emulsions decreased with NaCl (0.20 M). NaCl could shield the electrostatic adsorption between SDF and OPI, which would cause the complexes to separate and lower the EAI [51]. According to Chang et al. [35], NaCl could competitively bind water molecules and reduce the hydrophilicity of the sample molecules (i.e., salting-out effect), further modulating emulsifying properties. The flocculation phenomenon in sample emulsions was observed in Figure 6A. Compared to the blank group, the EAI of SDF-OPI decreased by 34.43% and that of untreated SDF-OPI decreased by 38.86%, which indicated that SDF-OPI was more salt-resistant than untreated SDF-OPI.

As seen in Figure 6A, the ESI of SDF-OPI was higher than that of untreated SDF-OPI after adding NaCl. Compared to the blank group, the ESI of SDF-OPI decreased by 31.70% and that of untreated SDF-OPI decreased by 56.47%. Hence, it was speculated that there were binding modes beyond electrostatic binding in the SDF-OPI that could improve its ESI.

#### 3.8.4. Thermal Stability Analysis

Since emulsions are frequently processed with heat treatment, heat resistance is one of the most important indicators of emulsification functions. The effect of heat treatment on the emulsifying properties of ultrasonic emulsions is shown in Figure 6B. Compared to the blank group, the EAI and ESI of SDF-OPI after heat treatment decreased by 31.00% and 9.48%, while the EAI and ESI of untreated SDF-OPI after heat treatment decreased by 35.19% and 21.50%, respectively. This was associated with the thermal denaturation of proteins, which could expose hydrophobic residues, reduce solubility, and lead to flocculation [51]. At the same time, heating could accelerate droplet thermal motion, facilitating aggregation, and may also induce SDF degradation into shorter fragments [35]. After the same heat treatment, the EAI and ESI of the SDF-OPI were significantly higher than that of the untreated SDF-OPI, SDF, and OPI (*p* < 0.05), indicating that the heat resistance and emulsification properties of the SDF-OPI were better.

### 3.9. Foamability and Foam Stability

Protein solutions are surface-active and can reduce the interfacial tension between water and air, thus providing some foamability. Polysaccharides help proteins disperse in solution, and dietary fibers with excellent hydrophilicity can also improve the stability of protein foams through thickening and gelling [52]. As seen in Figure 6C, the foamability and foam stability of SDF-OPI were higher than those of SDF and OPI (*p* < 0.05), while the difference between SDF-OPI and untreated SDF-OPI was not significant. Usually, the foamability of proteins is positively correlated with their hydrophobicity. The solubility of SDF-OPI was better than that of untreated SDF-OPI, inferring that the hydrophobicity of SDF-OPI was weaker, which affected its foaming performance.

## 4. Conclusions

This study showed that the complexation of soluble dietary fiber and protein from olives improved the solubility, thermal stability, emulsifying properties, and foaming properties of the protein, which was mainly attributed to the spatial site resistance effects of polysaccharides. The pH value during the complexation process affected the complexes, which was related to the isoelectric point of the protein. The results showed that the SDF-OPI complex formed at pH 5 had better properties, with a small particle size, low turbidity, and the greatest improvement in the solubility of OPI. The use of thermal–ultrasonic treatment significantly improved the complexation of SDF with OPI and exhibited better emulsifying properties compared to the conventional wet-heating technique. This enhancement was due to the capacity of intense ultrasonic waves to encourage protein unfolding and break down protein aggregates. Under acidic conditions, SDF and OPI were bound primarily by electrostatic interactions, assisted by hydrophobic interactions. Covalent binding between the two may also exist after thermal–ultrasonic treatment, which could explain its better hydrophilicity and thermal stability. In summary, the complexes are useful for expanding the application of olive proteins and soluble dietary fibers as functional ingredients in acid-dispersed systems.

## Figures and Tables

**Table 1:** Thermal characteristic parameters of OPI, SDF, untreated SDF-OPI, and the SDF-OPI complex.

Types	T_on_ (°C)	T_d_ (°C)	T_end_ (°C)	ΔH (J/g)
OPI	35.13 ± 0.09 ^d^	66.59 ± 0.43 ^d^	105.07 ± 0.74 ^d^	114.83 ± 0.98 ^c^
SDF	53.18 ± 0.11 ^a^	100.31 ± 0.89 ^a^	200.00 ± 0.00 ^a^	353.95 ± 3.67 ^a^
untreated SDF-OPI	48.84 ± 0.09 ^b^	75.81 ± 0.33 ^c^	120.39 ± 1.03 ^b^	112.45 ± 1.10 ^c^
SDF-OPI	38.67 ± 0.12 ^c^	76.28 ± 0.45 ^b^	117.49 ± 1.21 ^c^	172.33 ± 2.23 ^b^

All data represent means ± standard deviation, and the number of samples is *n* = 3. Mean values with different lowercase letters (^a,b,c,d^) in the same column are significantly different as determined by Fisher’s test (*p* < 0.05). T_on_: Onset denaturation temperature; T_d_: denaturation temperature; T_end_: endset denaturation temperature; ΔH: enthalpy changes.

## Data Availability

The original contributions presented in the study are included in the article, further inquiries can be directed to the corresponding author.

## References

[1] Eddaoukhi A., Berradi M., El Rhayam Y., Rissouli L., Grou M., El Yacoubi A., Bouraada K., Zerrouk M.H., El Bachiri A., Nassali H. (2023). Characterizing and optimizing adsorption for olive mill wastewater processing in Loukkos, Morocco. Environ. Monit. Assess..

[2] Aguilera-Huertas J., Parras-Alcántara L., González-Rosado M., Lozano-García B. (2024). Intercropping in rainfed Mediterranean olive groves contributes to improving soil quality and soil organic carbon storage. Agric. Ecosyst. Environ..

[3] Kocadağlı T., Yılmaz C., Gökmen V. (2024). Effects of fermentation and alkalisation on the formation of endocannabinoid-like compounds in olives. Food Chem..

[4] Nunes M.A., Palmeira J.D., Melo D., Machado S., Lobo J.C., Costa A.S.G., Alves R.C., Ferreira H., Oliveira M.B.P.P. (2021). Chemical Composition and Antimicrobial Activity of a New Olive Pomace Functional Ingredient. Pharmaceuticals.

[5] Dhingra D., Michael M., Rajput H., Patil R.T. (2012). Dietary fibre in foods: A review. J. Food Sci. Technol..

[6] Lee Y.H., Kalailingam P., Delcour J.A., Fogliano V., Thanabalu T. (2023). Olive-Derived Antioxidant Dietary Fiber Modulates Gut Microbiota Composition and Attenuates Atopic Dermatitis Like Inflammation in Mice. Mol. Nutr. Food Res..

[7] Speroni C.S., Bender A.B.B., Stiebe J., Ballus C.A., Ávila P.F., Goldbeck R., Morisso F.D.P., Silva L.P.d., Emanuelli T. (2020). Granulometric fractionation and micronization: A process for increasing soluble dietary fiber content and improving technological and functional properties of olive pomace. LWT.

[8] Akharume F.U., Aluko R.E., Adedeji A.A. (2021). Modification of plant proteins for improved functionality: A review. Compr. Rev. Food Sci. Food Saf..

[9] Rodríguez G., Lama A., Rodríguez R., Jiménez A., Guillén R., Fernández-Bolaños J. (2008). Olive stone an attractive source of bioactive and valuable compounds. Bioresour. Technol..

[10] Jha B., Singh N.P., Mishra A. (2012). Proteome Profiling of Seed Storage Proteins Reveals the Nutritional Potential of Salicornia brachiata Roxb., an Extreme Halophyte. J. Agric. Food Chem..

[11] Han Y., Zhu L., Zhang H., Liu T., Wu G. (2024). Characteristic of the interaction mechanism between soy protein isolate and functional polysaccharide with different charge characteristics and exploration of the foaming properties. Food Hydrocoll..

[12] Liu G., Li W., Qin X., Zhong Q. (2020). Pickering emulsions stabilized by amphiphilic anisotropic nanofibrils of glycated whey proteins. Food Hydrocoll..

[13] Zhao H., Wang S., Zhao G., Li Y., Liu X., Yang L., Zhu L., Liu H. (2022). Fabrication and emulsifying properties of non-covalent complexes between soy protein isolate fibrils and soy soluble polysaccharides. Food Funct..

[14] Wang K., Huang S., Xing S., Wu S., Li H., Zhong X., Na X., Tan M., Su W. (2023). On-Chip Precisely Controlled Preparation of Uniform Core–Shell Salmon Byproduct Protein/Polysaccharide Microcapsules for Enhancing Probiotic Survivability in Fruit Juice. J. Agric. Food Chem..

[15] Hu Y., Zhang Y., Xu J., Zi Y., Peng J., Zheng Y., Wang X., Zhong J. (2022). Fish gelatin-polysaccharide Maillard products for fish oil-loaded emulsion stabilization: Effects of polysaccharide type, reaction time, and reaction pH. LWT.

[16] Akhtar M., Ding R. (2017). Covalently cross-linked proteins & polysaccharides: Formation, characterisation and potential applications. Curr. Opin. Colloid Interface Sci..

[17] Nooshkam M., Varidi M. (2021). Physicochemical stability and gastrointestinal fate of β-carotene-loaded oil-in-water emulsions stabilized by whey protein isolate-low acyl gellan gum conjugates. Food Chem..

[18] Fan X., Li C., Shi Z., Xia Q., Du L., Zhou C., Pan D. (2024). Soy protein isolate–guar gum–goose liver oil O/W Pickering emulsions that remain stable under accelerated oxidation at high temperatures. J. Sci. Food Agric..

[19] Xu Y.-T., Liu L.-L. (2016). Structural and Functional Properties of Soy Protein Isolates Modified by Soy Soluble Polysaccharides. J. Agric. Food Chem..

[20] Li K., Wang J., Zhao P., Julian McClements D., Liu X., Liu F. (2024). Effect of ultrasound-assisted Maillard reaction on glycosylation of goat whey protein: Structure and functional properties. Food Chem..

[21] Baker P.W., Višnjevec A.M., Peeters K., Schwarzkopf M., Charlton A. (2023). Valorisation of waste olive pomace: Laboratory and pilot scale processing to extract dietary fibre. Clean. Circ. Bioecon..

[22] Gan J., Huang Z., Yu Q., Peng G., Chen Y., Xie J., Nie S., Xie M. (2020). Microwave assisted extraction with three modifications on structural and functional properties of soluble dietary fibers from grapefruit peel. Food Hydrocoll..

[23] Miranda C., Xu Q., Oehrle N.W., Islam N., Garrett W.M., Natarajan S.S., Gillman J.D., Krishnan H.B. (2019). Proteomic Comparison of Three Extraction Methods Reveals the Abundance of Protease Inhibitors in the Seeds of Grass Pea, a Unique Orphan Legume. J. Agric. Food Chem..

[24] Bilir G., Khalesi M., Cermeño M., FitzGerald R.J., Ekinci D. (2022). Extraction and Characterization of Protein Concentrates from Limpets (Patella vulgata) and Peptide Release Following Gastrointestinal Digestion. J. Agric. Food Chem..

[25] Chen K., Zhang M., Adhikari B., Wang M. (2022). Microencapsulation of Sichuan pepper essential oil in soybean protein isolate-Sichuan pepper seed soluble dietary fiber complex coacervates. Food Hydrocoll..

[26] Dong Z., Yu S., Zhai K., Bao N., Rashed M.M.A., Wu X. (2023). Fabrication and Characterization of Complex Coacervation: The Integration of Sesame Protein Isolate-Polysaccharides. Foods.

[27] Rezaei M., Nouri L., Daneshi M., Nafchi A.M., Nahidi F. (2022). Effect of salt concentration and drying temperature on functional properties of sesame (*Sesamum indicum* L.) meal protein isolate. J. Food Meas. Charact..

[28] Wang S., Liu X., Zhao G., Li Y., Yang L., Zhu L., Liu H. (2022). Protease-induced soy protein isolate (SPI) characteristics and structure evolution on the oil–water interface of emulsion. J. Food Eng..

[29] Wang S., Zhao H., Qu D., Yang L., Zhu L., Song H., Liu H. (2022). Destruction of hydrogen bonding and electrostatic interaction in soy hull polysaccharide: Effect on emulsion stability. Food Hydrocoll..

[30] Chen X.-W., Yin W.-J., Yang D.-X., Wan Z.-L., Ma C.-G., Yang X.-Q. (2021). One-pot ultrasonic cavitational emulsification of phytosterols oleogel-based flavor emulsions and oil powder stabilized by natural saponin. Food Res. Int..

[31] Liu Y., Liang Q., Liu Y., Rashid A., Qayum A., Ma H., Ren X. (2023). Effects of multi-frequency ultrasound on sodium caseinate/pectin complex: Emulsifying properties, interaction force, structure and correlation. Int. J. Biol. Macromol..

[32] Fernandez-Avila C., Gutierrez-Merida C., Trujillo A.J. (2017). Physicochemical and sensory characteristics of a UHT milk-based product enriched with conjugated linoleic acid emulsified by Ultra-High Pressure Homogenization. Innov. Food Sci. Emerg. Technol..

[33] Zou H., Zhao N., Li S., Sun S., Dong X., Yu C. (2020). Physicochemical and emulsifying properties of mussel water-soluble proteins as affected by lecithin concentration. Int. J. Biol. Macromol..

[34] Tan Y., Wannasin D., McClements D.J. (2023). Utilization of potato protein fractions to form oil-in-water nanoemulsions: Impact of pH, salt, and heat on their stability. Food Hydrocoll..

[35] Russell C., Zompra A.A., Spyroulias G.A., Salek K., Euston S.R. (2021). The heat stability of Rhamnolipid containing egg-protein stabilised oil-in-water emulsions. Food Hydrocoll..

[36] Alavi F., Chen L., Wang Z., Emam-Djomeh Z. (2021). Consequences of heating under alkaline pH alone or in the presence of maltodextrin on solubility, emulsifying and foaming properties of faba bean protein. Food Hydrocoll..

[37] Liu L., Fishman M.L., Hicks K.B., Kende M. (2005). Interaction of various pectin formulations with porcine colonic tissues. Biomaterials.

[38] Nakamura A., Maeda H., Corredig M. (2004). Competitive adsorption of soy soluble polysaccharides in oil-in-water emulsions. Food Res. Int..

[39] Guo Q., Li S., Du G., Chen H., Yan X., Chang S., Yue T., Yuan Y. (2022). Formulation and characterization of microcapsules encapsulating carvacrol using complex coacervation crosslinked with tannic acid. LWT.

[40] Feng J., Liu S., Sun N., Dong H., Miao L., Wang H., Tong X., Jiang L. (2024). Combining different ionic polysaccharides and pH treatment improved functional properties of soybean protein amyloid fibrils through structural modifications. Food Hydrocoll..

[41] Zha F., Yang Z., Rao J., Chen B. (2021). Correction to Gum Arabic-Mediated Synthesis of Glyco-pea Protein Hydrolysate via Maillard Reaction Improves Solubility, Flavor Profile, and Functionality of Plant Protein. J. Agric. Food Chem..

[42] Nawrocka A., Szymańska-Chargot M., Miś A., Wilczewska A.Z., Markiewicz K.H. (2016). Dietary Fiber-Induced Changes in the Structure and Thermal Properties of Gluten Proteins Studied by Fourier Transform-Raman Spectroscopy and Thermogravimetry. J. Agric. Food Chem..

[43] Li S., Hu N., Zhu J., Zheng M., Liu H., Liu J. (2022). Influence of modification methods on physicochemical and structural properties of soluble dietary fiber from corn bran. Food Chem. X.

[44] Yong Y.H., Yamaguchi S., Matsumura Y. (2006). Effects of Enzymatic Deamidation by Protein-Glutaminase on Structure and Functional Properties of Wheat Gluten. J. Agric. Food Chem..

[45] Makshakova O.N., Bogdanova L.R., Faizullin D.A., Ermakova E.A., Zuev Y.F. (2023). Sulfated Polysaccharides as a Fighter with Protein Non-Physiological Aggregation: The Role of Polysaccharide Flexibility and Charge Density. Int. J. Mol. Sci..

[46] Bi J., Sun Y., Pan D., Zhou C., Du L. (2024). Effect of ultrasound combined with psyllium husk powder on the structure and gel properties of goose myofibrillar protein. Process Biochem..

[47] Zhang Y., Zhao W., Yang R. (2015). Steam Flash Explosion Assisted Dissolution of Keratin from Feathers. ACS Sustain. Chem. Eng..

[48] Ai C., Zhao C., Guo X., Chen L., Yu S. (2022). Physicochemical properties of whey protein isolate and alkaline soluble polysaccharide from sugar beet pulp conjugates formed by Maillard reaction and genipin crosslinking reaction: A comparison study. Food Chem. X.

[49] Wan C., Yu S., Dang P., Gao L., Ge J., Li Y., Yang H., Yang P., Feng B., Gao J. (2023). Nitrogen regulates the synthesis of hydrophobic amino acids to improve protein structural and gel properties in common buckwheat. Int. J. Biol. Macromol..

[50] Fang H., Li J., Huo T., Niu Y., Yu L. (2021). Novel double cross-linked gels of soybean protein isolates and soluble dietary fiber from soybean coats with their functionalities. Food Hydrocoll..

[51] Chang L., Lan Y., Chen B., Rao J. (2023). Interfacial, and emulsifying properties nexus of green pea protein fractions: Impact of pH and salt. Food Hydrocoll..

[52] Li K.Y., Ye J.T., Yang J., Shao J.Q., Jin W.P., Zheng C., Wan C.Y., Peng D.F., Deng Q.C. (2023). Co-Extraction of Flaxseed Protein and Polysaccharide with a High Emulsifying and Foaming Property: Enrichment through the Sequence Extraction Approach. Foods.

